# Identifying potential regulators of JAGGED1 expression in portal mesenchymal cells

**DOI:** 10.1186/s13104-022-06058-4

**Published:** 2022-05-13

**Authors:** Teppei Nishino, Masaharu Yoshihara, Takahiro Nakayama, Takaho Tsuchiya, Saeko Tahara, Haruka Ozaki, Satoru Takahashi

**Affiliations:** 1 Department of Anatomy and Embryology, Faculty of Medicine, University of Tsukuba, Tsukuba, Japan; 2 Tsukuba Medical Center Hospital, Tsukuba, Japan; 3 Ph.D. Program in Humanics, School of Integrative and Global Majors, University of Tsukuba, Tsukuba, Japan; 4 Department of Primary Care and Medical Education, Faculty of Medicine, University of Tsukuba, Tsukuba, Japan; 5 College of Medicine, School of Medicine and Health Sciences, University of Tsukuba, Tsukuba, Japan; 6 Bioinformatics Laboratory, Faculty of Medicine, University of Tsukuba, Tsukuba, Japan; 7 Center for Artificial Intelligence Research, University of Tsukuba, Tsukuba, Japan; 8 Laboratory Animal Resource Center, 1-1-1 Tennodai, Tsukuba, Ibaraki 305-8575 Japan

**Keywords:** Delta-Notch signaling pathway, DoRothEA, EGR1, Intrahepatic biliary development, Regulon evaluations, SLUG, Single-cell RNA-seq, Smooth muscle actin, SOX2, Transcription factors

## Abstract

**Objective:**

Portal mesenchymal cells induce the epithelial differentiation of the bile ducts in the developing liver via one of the Delta-Notch signaling components, JAGGED1. Although this differential induction is crucial for normal liver physiology as its genetic disorder (Alagille syndrome) causes jaundice, the molecular mechanism behind JAGGED1 expression remains unknown. Here, we searched for upstream regulatory transcription factors of JAGGED1 using an integrated bioinformatics method.

**Results:**

According to the DoRothEA database, which integrates multiple lines of evidence on the relationship between transcription factors and their downstream target genes, three transcription factors were predicted to be upstream of JAGGED1: SLUG, SOX2, and EGR1. Among these, SLUG and EGR1 were enriched in ACTA2-expressing portal mesenchymal cells in two previously reported human fetal liver single-cell RNA-seq datasets. JAGGED1-expressing portal mesenchymal cells tended to express SLUG rather than EGR1, supporting that SLUG induced JAGGED1 expression. Together with the higher confidentiality of SLUG (DoRothEA level A) over EGR1 (DoRothEA level D), we concluded that SLUG was one of the most important candidate transcription factors upstream of JAGGED1. These results add mechanistic insights into the developmental biology of how portal mesenchymal cells support biliary development in the liver.

**Supplementary Information:**

The online version contains supplementary material available at 10.1186/s13104-022-06058-4.

## Introduction

Identification of the upstream regulators of key genes is one of the central issues in developmental biology. In the field of bile duct formation in the liver, one of the Delta-Notch signaling components, JAGGED1, is crucial, as understood by jaundice in patients with genetic abnormalities (Alagille syndrome) [1, 2]. One of the major complications of this disease is abnormalities in the bile ducts in the liver, often requiring liver transplantation. JAGGED1 is expressed in portal mesenchymal cells that are characterized by smooth muscle actin-associated genes such as TAGLN [3] and ACTA2 [4]. From an experimental point of view, TAGLN-Cre-mediated JAGGED1 deletion in portal mesenchymal cells caused significant jaundice [3], suggesting that portal mesenchymal cells express JAGGED1 to induce epithelial differentiation that occurs in their periphery between mouse embryonic Days 13.5 (E13.5) and E18.5 [5]. However, the molecular mechanism underlying the induction of JAGGED1 expression in portal mesenchymal cells remains unknown. As portal mesenchymal cells occupy a small percentage (less than 10%) of the total liver cells (discussed later), we considered that it would be difficult to identify potential upstream transcription factors using conventional biochemical methods.

Given this, we went on to identify the upstream transcription factor regulating the induction of JAGGED1 expression in portal mesenchymal cells using an established bioinformatics database called DoRothEA [6]. DoRothEA uses several lines of data to generate a table of combinations of specific transcription factors and their downstream targets (regulons) with the confidentiality of these combinations scored from A (highest confidence) to E (lowest confidence), depending on the amount of evidence supporting these interactions. This means that a DoRothEA level A interaction is supported by at least two curated sources. We used this database to identify potential upstream transcription factors regulating JAGGED1 expression. We then examined the validity of these results by analyzing two sets of previously reported human fetal liver single-cell RNA-seq data [7]. This strategy of narrowing down the potential transcription factors provides realistic insights into the regulation of a developmentary important gene component, JAGGED1.

## Main text

### Methods

We used R (R version 3.6.3; https://www.R-project.org/) to refer to DoRothEA.

We used the raw FASTQ files of single-cell RNA sequencing data from human fetal livers at Carnegie Stages (CS) 20 (GSM3906001) and 23 (GSM3906002), which correspond to E14.5 and E16, respectively. Both datasets were retrieved from the NCBI Gene Expression Omnibus (https://www.ncbi.nlm.nih.gov/geo/). We used CellRanger (v5.0.1) [8] to align the sequencing reads in the FASTQ files to the human reference (GRCh38-2020-A, 10x Genomics) and created a count matrix for subsequent analysis. We used Scanpy (v1.7.0) [9] for basic filtering and comparing the enrichment of specific genes between one cluster and the others. Cells with high percentages of mitochondrial (> 0.1) or hemoglobin gene (> 0.1) expression and cells with a low percentage of ribosomal gene expression (< 0.05) were excluded from subsequent analysis. Any doublets identified by Scrublet (v0.2.3) were also excluded. Then, highly variable genes (n = 3019 and 2603 for CS20 and 23 datasets, respectively) across the single-cell datasets (n = 7028 and 8020 for CS20 and 23 datasets, respectively) were identified using the “sc.pp.highly_variable_genes” function and then subjected to dimension reduction with principal component analysis (“sc.tl.pca” function) and UMAP (“sc.tl.umap” function) before clustering (Leiden method). Single-cell RNA sequencing analysis was completed in Python 3.6.13 in an Ubuntu 20.04 LTS environment. We examined differences in gene expression within these clusters using the Mann–Whitney U test, with these outcomes described as FDR-adjusted p values in the Results section.

### Results

#### SLUG, SOX2, and EGR1 as potential regulators of JAGGED1

We first used DoRothEA to identify several potential regulators of JAGGED1 in portal mesenchymal cells and focused on SLUG (also known as SNAI2), SOX2, and EGR1, which are listed in the DoRothEA database for their association with JAGGED1 (levels A, B, and D, respectively) [6]. There were no candidate transcription factors with DoRothEA levels A or B other than SLUG and SOX2. EGR1 may be important because it was expressed in portal mesenchymal cells (discussed later), whereas the other transcription factors of DoRothEA level C or D, which are listed in Additional file 1, were not enriched in portal mesenchymal cells in either the CS20 or 23 datasets.

#### Identification of ACTA2-expressing portal mesenchymal cells

**Fig. 1:**
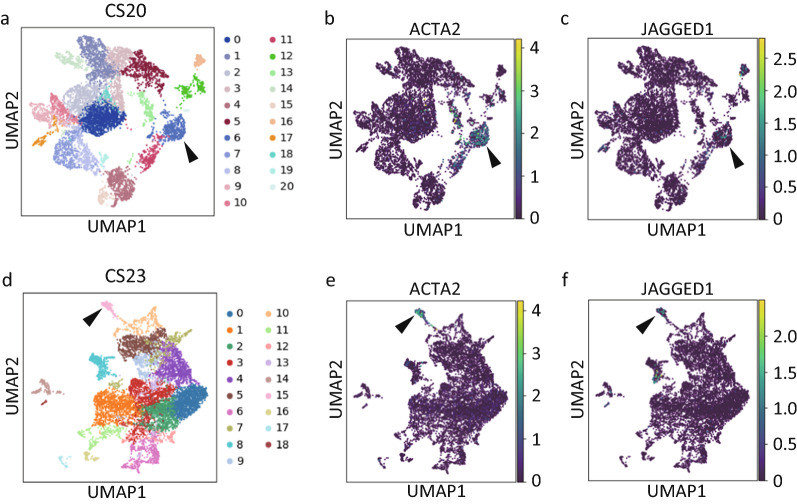
Identification of portal mesenchymal cells in human fetal liver single-cell RNA-seq data. **a** UMAP plot of CS20 human fetal liver single-cell RNA-seq data. **b** Cluster #6 in **a** was enriched for ACTA2 and contained portal mesenchymal cells (arrowhead). **c** Cluster #6 contained JAGGED1-expressing cells (arrowhead). **d** UMAP plot of CS23 human fetal liver single-cell RNA-seq data. **e** Cluster #15 in **d** was enriched for ACTA2 and contained portal mesenchymal cells (arrowhead). **f** Cluster #15 contained JAGGED1-expressing cells (arrowhead)

Next, we searched for TAGLN-expressing portal mesenchymal cells in previously reported single-cell RNA-seq data from human fetal liver tissues of the CS20 and 23 datasets as described in the Methods section. Unfortunately, those cells occupied only 3.54% and 1.11% of the total cells of the CS20 and 23 datasets, respectively. Therefore, we alternatively searched for ACTA2-expressing portal mesenchymal cells in the CS20 and 23 datasets. In contrast to TAGLN, ACTA2-expressing cells occupied 5.92% and 5.76% of the total cells in the CS20 and 23 datasets, respectively, and we used ACTA2 as a portal mesenchymal cell marker gene. For the CS20 dataset, we conducted cell clustering as described in the Methods section, and that evaluation produced 21 clusters (Fig. 1a), one of which (cluster #6) contained 466 cells (6.63% of total cells) with increased ACTA2 (p = 8.21 × 10^–24^) expression (Fig. 1b), identifying them as portal mesenchymal cells. As expected, this cell population expressed a relatively high level of JAGGED1 (log fold-change = 2.30, p = 0.439) (Fig. 1c). For the CS 23 dataset, these evaluations produced 19 clusters (Fig. 1d), one of which (cluster #15) contained 129 cells (4.96% of total cells) with increased ACTA2 (p = 1.80 × 10^–28^) expression (Fig. 1e), identifying them as portal mesenchymal cells. Indeed, this cell population expressed a significantly high level of JAGGED1 (p = 0.0384) (Fig. 1f).

#### Evaluation of the validity of SLUG, SOX2, and EGR1

**Fig. 2:**
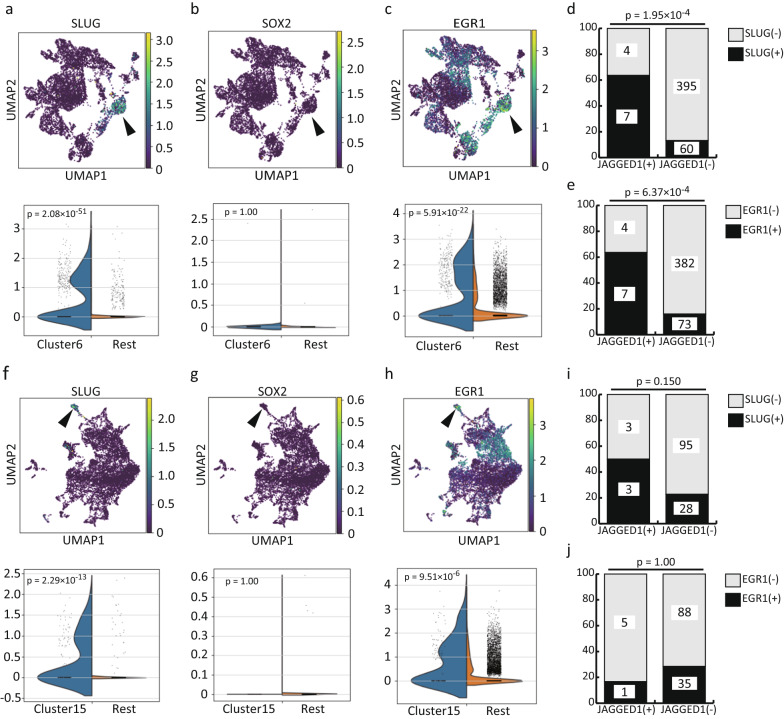
Examination of enrichment of potential regulators in portal mesenchymal cells. **a** SLUG was enriched in cluster #6 of the CS20 dataset (FDR-adjusted p value = 2.08 × 10^–51^). **b** SOX2 was not enriched in cluster #6 of the CS20 dataset (FDR-adjusted p value = 1.00). **c** EGR1 was enriched in cluster #6 of the CS20 dataset (FDR-adjusted p value = 5.91 × 10^–22^). **d** Comparison of JAGGED1 or SLUG expression in the cells of cluster #6 of the CS20 dataset as evaluated by Fisher’s exact test. Note that the percentage of SLUG-expressing cells was significantly higher in JAGGED1-expressing cells than in JAGGED1-nonexpressing cells (p = 1.95 × 10^–4^). **e** Comparison of JAGGED1 or EGR1 expression in the cells of cluster #6 of the CS20 dataset as evaluated by Fisher’s exact test. Note that the percentage of EGR1-expressing cells was significantly higher in JAGGED1-expressing cells than in JAGGED1-nonexpressing cells (p = 6.37 × 10^–4^). **f** SLUG was enriched in cluster #15 of the CS23 dataset (FDR-adjusted p value = 2.29 × 10^–13^). **g** SOX2 was not enriched in cluster #15 of the CS23 dataset (FDR-adjusted p value = 1). **h** EGR1 was enriched in cluster #15 of the CS23 dataset (FDR-adjusted p value = 9.51 × 10^–6^). **i** Comparison of JAGGED1 or SLUG expression in the cells of cluster #15 of the CS23 dataset as evaluated by Fisher’s exact test. Note that the percentage of SLUG-expressing cells was higher in JAGGED1-expressing cells than in JAGGED1-nonexpressing cells, although the difference was not statistically significant (p = 0.150). **j** Comparison of JAGGED1 or EGR1 expression in the cells in cluster #15 of the CS23 dataset as evaluated by Fisher’s exact test. Note that the percentage of EGR1-expressing cells was lower in JAGGED1-expressing cells than in JAGGED1-nonexpressing cells, although the difference was not statistically significant (p = 1.00)

Then, we examined the expression of SLUG, SOX2, and EGR1 in portal mesenchymal cells (clusters #6 and #15 of the CS20 and 23 datasets, respectively). For the CS20 dataset, the cells in cluster #6 abundantly expressed SLUG (p = 2.08 × 10^–51^) (Fig. 2a) and EGR1 (p = 5.91 × 10^–22^) (Fig. 2c) but not SOX2 (p = 1.00) (Fig. 2b). We further evaluated the expression of JAGGED1, SLUG, and EGR1 in the cells of cluster #6 of the CS20 dataset. This evaluation showed that 63.6% of JAGGED1-expressing cells also expressed SLUG (7 out of 11 cells), while 13.2% of JAGGED1-nonexpressing cells expressed SLUG (60 out of 455 cells) (p = 1.95 × 10^–4^, Fisher’s exact test) (Fig. 2d). Additionally, although 63.6% of JAGGED1-expressing cells also expressed EGR1 (7 out of 11 cells), only 16% of JAGGED1-nonexpressing cells expressed EGR1 (73 out of 455 cells) (p = 6.37 × 10^–4^, Fisher’s exact test); hence, we considered that both SLUG and EGR1 remained potential regulators (Fig. 2e).

For the CS23 dataset, the cells in cluster #15 abundantly expressed SLUG (p = 2.29 × 10^–13^) (Fig. 2f) and EGR1 (p = 9.51 × 10^–6^) (Fig. 2h) but not SOX2 (p = 1.00) (Fig. 2g). We further evaluated the expression of JAGGED1, SLUG, and EGR1 in the cells of cluster #15. This evaluation showed that 50.0% of JAGGED1-expressing cells also expressed SLUG (3 out of 6 cells), while only 22.8% of JAGGED1-nonexpressing cells expressed SLUG (28 out of 123 cells) (p = 0.150, Fisher’s exact test) (Fig. 2i). In contrast, although 16.7% of JAGGED1-expressing cells also expressed EGR1 (1 of 6 cells), as many as 28.5% of JAGGED1-nonexpressing cells expressed EGR1 (35 of 88 cells) (p = 1.00, Fisher’s exact test) (Fig. 2j). Therefore, together with high confidentiality (DoRothEA level A), we concluded that SLUG is a preferable candidate regulator of JAGGED1 expression in portal mesenchymal cells.

### Discussion

This study was designed to identify the upstream transcription factor regulating JAGGED1 expression in portal mesenchymal cells. Our evaluations found SLUG to be a central candidate for this regulatory role, as this protein is strongly expressed during the time frame associated with epithelial differentiation into portal mesenchymal cells. We considered that insignificant JAGGED1 expression in portal mesenchymal cells at CS20 was because this time point was relatively early to allow the expression of the upstream regulator, SLUG, rather than JAGGED1.

SLUG reportedly acts as a transcriptional suppressor, as previously observed during the downregulation of E-cadherin expression in breast cancer [10]. However, the results of the biochemical analysis of the induction of fatty acid synthase in the liver suggest that SLUG can act as an activator [11]. Additionally, an in vitro study using a human breast cancer cell line (MCF7) showed that SLUG overexpression or siRNA-mediated suppression leads to increased or decreased JAGGED1 expression, respectively, suggesting that SLUG expression is positively correlated with JAGGED1 expression in this cell line [12]. Although this observation, along with the absence of SLUG binding sites in the JAGGED1 promoter region according to DoRothEA [6], implies that SLUG does not directly regulate JAGGED1 expression, we propose that SLUG is located upstream of JAGGED1.

EGR1 is also a potential regulator enriched in mesenchymal cells (cluster #6 of the CS20 dataset and cluster #15 of the CS23 dataset). This transcription factor was reported to induce JAGGED1 expression in *Leishmania donovani*-infected bone marrow macrophages [13]. However, owing to its low confidentiality (DoRothEA level D) and scarce expression in JAGGED1-expressing portal mesenchymal cells in the CS23 dataset, we consider SLUG to be a potential regulator over EGR1.

### Conclusion

SLUG is the candidate regulator of JAGGED1 in portal mesenchymal cells.

## Limitations

First, further in vivo analysis is necessary to examine the biochemical properties of portal mesenchymal cells to determine the relationships between transcription factors and their regulons. Second, this study does not suggest that SLUG is the sole candidate for the upstream regulation of JAGGED1 expression because we only evaluated transcription factors according to DoRothEA. Third, four transcription factors (ESR1, HNB1B, PRDM14, and TFAP2C) were not examined for their enrichment because they were not differentially expressed genes in either CS20 or 23 datasets. Finally, we note that the molecular signature of portal mesenchymal cells is not fully characterized, and hence, other transcription factors may be listed as potential regulators when we use other marker genes for portal mesenchymal cells. However, we consider our work significant in that this is the first report on the molecular regulation of JAGGED1 expression in portal mesenchymal cells, which is important for normal liver physiology.

## Supplementary Information

**Additional file 1.** This table shows all the transcription factors listed in DoRothEA that have an association with JAGGED1 as their target gene. SNAI2 (SLUG), SOX2, and SOX13 had DoRothEA levels of A, B, and C, respectively. The other 32 transcription factors had DoRothEA levels of D. The line number indicates the corresponding positions in DoRothEA.

## Data Availability

Codes of the single-cell RNA sequencing analyses are available from our GitHub repository (https://github.com/Teppei-Nishino/Slug).

## Abbreviations

ACTA2: Actin alpha 2
CS: Carnegie stage
NCBI: National Center for Biotechnology Information
SNAI2: Snail family transcriptional repressor 2
TAGLN: Transgelin
UMAP: Uniform manifold approximation and projection
