# Supplementary material for: Identifying potential regulators of JAGGED1 expression in portal mesenchymal cells
Source: BMC Res Notes. 2022 May 13;15:172. doi: 10.1186/s13104-022-06058-4 (PMC9102744; doi:10.1186/s13104-022-06058-4)
Supplement: Supplementary file 1 — Additional file 1. This table shows all the transcription factors listed in DoRothEA that have an association with JAGGED1 as their target gene. SNAI2 (SLUG), SOX2, and SOX13 had DoRothEA levels of A, B, and C, respectively. The other 32 transcription factors had DoRothEA levels of D. The line number indicates the corresponding positions in DoRothEA. [file 13104_2022_6058_MOESM1_ESM.doc]

| Line No. | Gene Name | Dorothea Level |
| --- | --- | --- |
| 208883 | SNAI2 | A |
| 212040 | SOX2 | B |
| 210433 | SOX13 | C |
| 8972 | BCL11A | D |
| 15891 | CEBPA | D |
| 31366 | CTCF | D |
| 40822 | DNMT3B | D |
| 50272 | E2F4 | D |
| 60334 | EGR1 | D |
| 67169 | ELF3 | D |
| 72015 | ESR1 | D |
| 86819 | ETV4 | D |
| 101437 | FOXA1 | D |
| 111059 | FOXP1 | D |
| 116843 | GATA2 | D |
| 128778 | GLI2 | D |
| 132522 | HNF1B | D |
| 136826 | HNF4A | D |
| 145331 | IRF1 | D |
| 151494 | KLF4 | D |
| 152453 | KLF6 | D |
| 163450 | MITF | D |
| 173257 | NANOG | D |
| 191750 | PPARG | D |
| 193896 | PRDM14 | D |
| 197306 | RBPJ | D |
| 198050 | REL | D |
| 198430 | RELA | D |
| 203634 | RUNX3 | D |
| 218303 | SPI1 | D |
| 226636 | STAT1 | D |
| 239748 | TCF12 | D |
| 248945 | TFAP2C | D |
| 258747 | TP53 | D |
| 260468 | TP63 | D |

**Table Legend**

This table shows all the transcription factors listed in DoRothEA that have an association with JAGGED1 as their target gene. SNAI2 (SLUG), SOX2, and SOX13 had DoRothEA levels of A, B, and C, respectively. The other 32 transcription factors had DoRothEA levels of D. The line number indicates the corresponding positions in DoRothEA.
